# Five Applications of Narrative Exposure Therapy for Children and Adolescents Presenting With Post-Traumatic Stress Disorders

**DOI:** 10.3389/fpsyt.2020.00019

**Published:** 2020-02-19

**Authors:** Mina Fazel, Hannah J. Stratford, Eleanor Rowsell, Carmen Chan, Helen Griffiths, Katy Robjant

**Affiliations:** ^1^ Department of Psychiatry, University of Oxford, Warneford Hospital, Oxford, United Kingdom; ^2^ Children’s Psychological Medicine, Oxford Children’s Hospital, Oxford University Hospitals NHS Foundation Trust, Oxford, United Kingdom; ^3^ Highfield Adolescent Unit, Oxford Health NHS Foundation Trust, Warneford Hospital, Oxford, United Kingdom; ^4^ The Sue Nicholls Centre, Oxford Health NHS Foundation Trust, Aylesbury, United Kingdom; ^5^ Horizon (Supporting Young People and Families Affected by Sexual Harm), Oxford Health NHS Foundation Trust, Oxford, United Kingdom; ^6^ Vivo International, Konstanz, Germany

**Keywords:** trauma, post-traumatic stress disorder, children, adolescents, treatment

## Abstract

Narrative exposure therapy (NET) is an individual therapeutic approach that has an emerging evidence base for children. It was initially trialed with refugee and asylum seeking populations, in low, middle and high-income settings, utilizing either lay or professional therapists. The results of treatment trials for PTSD in refugee children with NET (or the child “KIDNET” adaptation) demonstrates how this is an effective intervention, is scalable and culturally dexterous. This paper describes, in five cases from clinical practice settings, the applicability of NET into broader, routine practice. The cases outlined describe the use of NET with adolescents with: autism spectrum disorders, psychotic symptoms, and intellectual disabilities; histories of forced abduction into child soldiering; complex physical health problems needing multiple interventions; and victims of childhood sexual abuse. The cases are discussed with regards to how the NET lifeline facilitated engagement in treatment, practical adaptations for those with intellectual disabilities and how NET, with its relatively short training for health professionals, can be modified to different contexts and presentations. The importance of improving access to care is discussed to ensure that young people are supported with their most complex and disruptive memories.

## Introduction

The sequelae of exposure to extremely distressing events, including the development of post-traumatic stress disorder (PTSD) remain an area of poor service provision across low, middle, and high-income nations, especially for child populations. Epidemiological studies have consistently shown that exposure to a range of potentially traumatic events is common in children and that symptoms of post-traumatic stress are also common although most do not actually develop PTSD (1, 2). It is increasingly recognized that PTSD, as a result of multiple traumatic events, may lead to a more complex presentation of PTSD than PTSD as a result of a single traumatic event (3). Children who have experienced multiple traumatic events or have a history of anxiety are at the greatest risk of developing PTSD and these experiences also have strong links with later anxiety and depression (2). It is therefore essential to improve access to treatments for PTSD in children and adolescents.

The core components of childhood PTSD, according to the Diagnostic and Statistical Manual of Mental Disorders, 5th Edition (DSM 5) categorization, include intrusive thoughts or memories, avoidance of trauma-related stimuli, negative trauma-related cognitions, a persistent negative emotional state, and exaggerated startle response (1, 4). Similarly, in the International Classification of Diseases 11th Revision (ICD-11), complex PTSD is described with similar core symptoms of re-experiencing, avoidance, and a sense of current threat alongside disturbances of self-organization which can include affect dysregulation, negative self-concept, and disturbances in relationships (5). These presenting features, in conjunction with primary caregivers often exposed to the same experiences (6–8), raise additional complexities in getting children with PTSD to enter and remain in treatment. For example, a core component of the disorder being treated is avoidance of any reminder of the original traumatic event. Therefore the individual is likely to be aware that exposure to these difficult memories is a crucial component of treatment, thus making entering treatment even more difficult (9). Parents might have similar concerns and believe they are protecting their children (and possibly themselves) by choosing not to attend a therapeutic intervention that will encourage reminders of the original traumatizing events (9, 10).

### Narrative Exposure Therapy

Narrative exposure therapy (NET) is an evidence-based intervention to treat those who have PTSD and have experienced multiple potentially traumatic events; originally developed by Neuner, Schauer, and Elbert (11–13). Although trauma-focused cognitive behavior therapy (TF-CBT) has the largest evidence-base for PTSD, these trials are not necessarily conducted on those who have experienced multiple traumatic events (14). NET is one of the 2018 National Institute for Health and Care Excellence (NICE) recommended interventions for the treatment of adult PTSD (15) with the large number of NET studies categorized and analyzed under the umbrella of TF-CBT interventions. There are, however, differences in the methodology of NET when compared to TF-CBT as in NET all the significant events are explored in detail and in chronological order taking a “lifeline” approach. Most of the other studies to systematize the impact of NET have specifically collated NET trials and not in combination with TF-CBT (15–18).

NET is a short-term, manualized, individual intervention which focuses on the core premise that during a traumatic event in which there was a physiological alarm response, these memories are encoded in a non-optimal manner (19). In brief, it is believed that the poor encoding of traumatic memories is, in an over-simplified explanation, as a result of the alarm response leading to an over functioning of the amygdala coupled with a decrease in functioning of the hippocampus. Thus, when triggered, “hot” memories for traumatic events (including the emotional, cognitive, sensory, and physiological experiences of the event) are more likely to be experienced in the absence of the temporal and spatial contextual information (cold memory) associated with the event. Although the neurological processes involved are highly complex (20, 21), these disturbances can, for example, lead to the subjective experience of a “flashback”—when the event is re-experienced in the here and now. In PTSD, this can lead to a fear of encountering any possible memories and triggers of the original event. Through the process of NET, the traumatic events are placed in chronological order as the memories are re-processed through a slow and detailed re-living, connecting the hot and cold memory together with additional awareness and recognition of the wider context and the meaning of these events for the individual. Furthermore, NET aims to repair the emotional associations of the original events through the relational re-living that takes place during treatment.

In brief, the treatment involves an initial “lifeline” to be mapped, often using a rope with “stones” to denote traumatic and negative events and flowers to mark happier or positive events. Following this session, the narrative exposure begins and continues to the end of treatment. Traumatic events are explored slowly, with questioning of multiple sensory, cognitive, emotional, and physiological information, with active linking to the “cold” memory with contextual information that, due to the high physiological arousal levels, were poorly encoded at the time of the trauma. This is then written in detail by the therapist and read at the beginning of the next session to ensure content is correct, to enable some more questions to be asked about gaps in the narrative and also to expose the young person, a second time, to the original traumatic event. In NET, the therapist does not remain neutral, but rather acknowledges the human rights abuses that the individual might have experienced (22). The design of the treatment for multiple traumas and its human rights focus, together with new developments involving family members, renders the approach potentially applicable to children traumatized in a range of circumstances who may present in different healthcare contexts (12).

There have only been five trials to date using NET to treat PTSD in child populations, predominantly conducted in low and middle income settings or for refugee and asylum seeker populations (23–27). Peltonen and Kangaslampi (23) conducted a multicenter randomized controlled trial (RCT) of 50 9–17 year olds in Finland who were refugees or who had PTSD as a result of family violence. In this study, only those treated with NET, compared with treatment as usual, had a clinically significant improvement in their PTSD symptoms. This is consistent with results from other studies and case reports (11, 28, 29), which support the use of NET for treating symptoms of PTSD in those who have experienced multiple traumatic events, it has low dropout rates indicating some degree of acceptability for those with PTSD and significant improvements in PTSD symptoms both at the end of treatment and in follow-up (11, 16, 17, 30). The NET studies in children (KIDNET) have, however, been of lower quality than those in adult populations. A recent meta-analysis of NET in adult populations was able to combine results from 16 RCTs and showed that although the treatment effects are significant when compared to non-active controls, they were comparable to active PTSD control treatments. Of note, treatment effects were also significant for treatment of depression and advancing age predicted better results (18).

There has been considerable interest in clinical services to use NET for treating PTSD, as it has a relatively short training for mental health professionals, and treatment is often completed in 8 to 10 sessions. For example, between 2005 and 2019, the authors alone have been involved in training over 500 mental health and allied professionals in the UK in NET. This started with a European Refugee Fund (ERF) “Multi-Centre NET-work Capacity-building” Project (JAI/2004/ERF/090). Only a minority of the trained practitioners are working with refugee and asylum seeker populations, and most are working in generic services. These practitioners are increasingly using NET as a treatment for individuals who have been exposed to multiple traumatic events. As a result of increasing interest in this area, we describe here the use of NET and its adaptation to five very different groups of young people with more complex PTSD and share the principles of practice and some of the ways it has been adapted to these situations. The aim of this paper is to encourage practitioners to explore opportunities to treat child populations with PTSD, especially as they poorly access mental health care (9, 14). The cases below have been used as the young people gave consent for their cases to be shared. Any identifying information has been changed.

## Five Case Examples

### Autism Spectrum Disorder and Psychosis

NET was adapted to treat a 15 year old female on the autism spectrum who had experienced both physical and sexual abuse and was an in-patient in an adolescent psychiatric unit. Her presentation had many psychotic features but during treatment, it became apparent that PTSD was the main driver of her psychotic symptoms (31, 32). Her poor expressive and receptive language, along with behavioral outbursts and agitation might have further complicated the presentation and made it more difficult to identify the traumatic component of her symptoms. For example, she reported seeing the abuser as a “real” vision, but with novel content leading to a belief that the abuser was in her home and cooperating with a family member and staff in the unit.

There are some components of NET that make it potentially suited to treating PTSD for those who are on the autism spectrum. As NET focuses primarily on the description of the original memory rather than also including associated interpretations and elaborated meta-level meanings of the events, it can potentially carry a lower cognitive load making it easier for someone with borderline intellectual disability and/or difficulties in expressive and receptive language.

The NET required some adaptations due to additional difficulties in the adolescent’s cognitive and social communication needs. The adaptations included spending more time exploring each “stone.” For example, with many individuals, the “stone” or traumatic event might need to be explored on one or two occasions in great detail, but in this case, multiple exposures were needed until the physiological arousal levels were lower and recollection of the events was clearer. She needed repetition of the rationale and additional time to process information. The NET was further adapted by using written prompt questions so she knew what questions to expect during sessions—for example, the core therapeutic questions asking about a range of current symptoms were written on sticky notes that could be carried all the time and provided some comfort that no unexpected questions would be asked. There were also written rationale statements explaining the process of NET to support engagement that she kept and could remind herself of before sessions. Finally, the whole treatment was conducted while walking up and down a corridor which helped to reduce dissociation and distress, and provided additional sensory coping strategies. This was perceived by the adolescent as a less intense setting than a clinic room and facilitated engagement as she did not like sitting in a room with a “therapist” just focusing on her and her experiences. Overall, the ward staff noted there was a reduction in self-harm and aggressive incidents, and a reduction in reliving symptoms.

On reflection, there were components of the psychotic symptoms that through exploration in NET were formulated to be poorly encoded memories contributing to PTSD. What was evident early in the treatment was how this young person found social interactions difficult, especially negative interactions with peers. This contributed to her experience of multiple traumas involving many negative events, a perception of ongoing bullying and other peer related difficulties—for example in the lifeline session, she placed a large number of stones throughout her life, with many events at school being highlighted. This was possibly because of her underdeveloped social theory of mind and impaired social skills, as has been described in autism spectrum disorders (33). She therefore found it difficult to appreciate different perspectives about events, and for example had a strong memory of being hit with a hockey stick at school recorded as a traumatic stone. The adolescent had recall and recognition difficulties, and low ability for new learning and so potentially perceived her memories as qualitatively different as well as often being difficult to access.

### Childhood Sexual Abuse

NET was used to treat a 14 year old female in an out-patient setting, who was sexually abused by her mother’s partner over a number of years. As is common for children who have experienced sexual abuse, this occurred within a context of other traumatic and distressing physical and emotional abuse and neglect, and within a chaotic home environment (34). At the time of referral, she was living in a stable foster placement with a number of professionals involved in her care. As a consequence of her early upbringing, she found it difficult to accept care from adults and was often mistrusting of others’ intentions. This was also present in her peer relationships. She used self-harm as a way of coping with high levels of emotion and, at assessment, she said that she often found herself becoming extremely angry for no reason. Upon formulating, we recognized that many of these difficulties were triggered by reminders of her past experiences, which were compounded by frequent flashbacks and nightmares. Her school attendance was low as she felt that school staff did not understand her or her past experiences.

She was given a choice between NET and TF-CBT. She chose NET as she felt that she had never had the chance to talk through all her experiences together. There have been two trials of NET in adults with PTSD with comorbid personality disorder, which have demonstrated a considerable reduction in symptoms of both PTSD and borderline personality disorder (35, 36). Given this patient’s self-harm behavior and difficulty with her emotional regulation and relationships, it was decided that NET might be able to address the breadth of her symptoms and needs, informed by components of these studies.

The NET protocol was adapted to ensure that the network of adults around her was included in the psychoeducation and treatment plan as it was important to ensure safeness and consistency in the system surrounding her (37). This involved additional meetings with her social worker, welfare officer at school, and foster carer to explain the process of therapy and to discuss how to best support her through the process, particularly when narrating a stone. After completing the lifeline, she decided to share her lifeline with those involved in her care. For her, this exercise, in itself, helped her contextualize the multiple trauma she had experienced and voice her story. She also felt that, after doing this, her school had a better understanding of what she had been through and were better able to think together with her foster carer about how to make school more manageable. Her foster carer attended the end of each session so that she could share and discuss as much as she wanted about the stone that had just been explored. She felt that this further helped those around her understand her experiences and to think about how to, jointly, manage the upcoming week and encourage attendance at the next treatment session.

On reflection, the young person responded well to the adapted NET, which included involving the professionals in her care and to bring in her foster carer at the end of each session. By the end of 12 sessions, her PTSD symptoms had reduced greatly, which also led to a reduction in self-harm and anger outbursts. Furthermore, her school attendance improved, as she felt those at school understood her better and were able to discuss and agree a coherent plan for managing any expressions of distress that might arise. There was also less conflict reported at home. For her, the collaborative approach involving individuals from the different systems understanding her better and supporting her, contributed to a sense of containment and safeness while undergoing NET.

### Child Soldier

NET was used to treat a series of child soldiers in the Democratic Republic of Congo (DRC) (38). Some children have not only been the victims of human rights abuses, but have also perpetrated them. Robjant *et al*. describe the treatment of 100 female former child soldiers abducted during the M23 war in Eastern DRC (38). This case was typical of those seen. A 17 year old presented for out-patient treatment. Abducted during a village attack, aged 12, she was taken with her male and female peers by a much-feared armed group. Within 24 hours she was raped, and later was forced to torture and kill some of her peer abductees. Thus began her “training” as a fighter which involved extreme physical brutality. At night, unlike the other girls who were afforded the title of “wife of soldier” and regularly raped by one person, she was “unassigned” and thus visited frequently by different men and groups of men. This afforded her a lower social status, as well as a likely increased trauma exposure, and as such she attempted to increase her status (and life expectancy) by being a better “fighter” using increasingly violent and deadly means (38).

Once freed back into the community she was using violence against others to reduce her own exposure to both her PTSD symptoms and extreme feelings of low self-worth. At the time of assessment, she was actively involved in the “set up” of females in the community to be raped by males “so that its just not me,” and was also involved in multiple incidents of physical violence against community members. Some of these acts of violence appeared to be instrumental aggression or related to general hyperarousal, but others were directly related to violence perpetration for pleasure—known as “appetitive aggression” (39). There is evidence that appetitive aggression is protective against PTSD symptoms and thus a protective factor for those living in unstable contexts (40, 41). In addition, she was self-medicating with alcohol when she was able to obtain it.

The treatment of this case involved an adaption of NET for perpetrators (FORNET) (38, 42), for example by marking acts of perpetration on the lifeline with “sticks.” Other adaptations include holding group sessions as part of the treatment which include: learning skills to manage affect regulation especially targeting anger; motivational work in avoiding violence; problem solving to help manage stigma and prejudice from community members (who often feared the returning abductees and sought to isolate and reject them); and active peer support through a buddying system (38).

Like others in the trial, this young person responded well to the adapted version of NET which involved only five individual exposure sessions. These sessions had a focus on re-processing traumatic events in which the traumatized individual had played different roles, at times as a perpetrator of events and other times not. Typically the lifeline showed large numbers of traumatic events, many of which also included perpetration of violence, and so in order to fit with the context (requiring low resource input) priority was afforded to events which were of different types, in differing contexts and which were related to symptoms (38). As was typical of the wider treatment cohort in the RCT, PTSD and depressive symptoms were reduced to the extent that she no longer met criteria for a diagnosis of either condition, appetitive aggression was reduced, but more importantly she had fewer episodes of actual violent behavior. In this particular case her alcohol use significantly reduced, in keeping with current theorizing about the role of trauma in substance misuse and the importance of trauma treatments for those who have substance misuse problems (43).

### Facilitating Engagement With Treatment

A 17 year old male was first referred to child mental health services for urgent multi-disciplinary input following initial support from the local mental health crisis team. There was a history of known trauma over a long period of time and his father was serving a prison sentence for abusing a sibling. Input included weekly individual therapy for initial diagnoses of severe depression, anxiety, and an eating disorder. After 3 months, the additional diagnosis of PTSD was established, and the young person chose NET over TF-CBT because although he was wary of interventions, he liked the idea of doing a lifeline and so he initially only agreed to attend the lifeline session.

NET was therefore adapted so that he could receive a first lifeline session before he committed to further treatment in the knowledge that as a potential stand-alone session it would not treat his PTSD. It did however contribute to a shared understanding and formulation. Although he was initially cautious about further treatment as avoidance of any reminders of his traumatic experiences was so strong, he decided to continue with NET after 3 months. Following completion of NET, he explained that he had not felt secure enough to “open up” at the beginning of the treatment as talking about his PTSD symptoms was more difficult than addressing depression or anxiety, and hence he needed longer to develop a trusting therapeutic relationship.

While he had experienced multiple and varied traumatic experiences from age 7 to 16, the visual representation of these as a lifeline helped illustrate how a number of stones related to the same events, the impact of which had not been fully appreciated. The trigger for this specific trauma was a current one, relating to a family member still living in his home. The treating team were then able to enhance the support offered by incorporating components to consider safeguarding and support within his home as well as treating the PTSD. The subsequent NET intervention was successful in reducing all reported symptoms of PTSD monitored by changes on the Child PTSD Symptom Scale (CPSS) and Impact of Events Scales supporting the clinically-observed and reported improvements (44, 45).

Reflecting on his experience of NET afterwards he felt that the initial task of completing the lifeline had helped his decision to proceed with NET. It was a non-threatening and relatively simple way of introducing NET so the young person could make a more informed decision about further treatment. He also, later, described this session as cathartic. As is not uncommon, it took him a few months until he felt able to commence the full NET, by which time his mood and risk had stabilized and regular sessions could be arranged around his availability. The visual representation of traumatic experiences in the lifeline led to a greater understanding for both the young person and therapist which then guided further interventions in the mental health service.

### Chronic Health Condition Needing Multiple Interventions

A 15 year old female who had had multiple cardiac surgeries from a young age had been seen by the hospital’s psychological medicine service for years of support following her operations as well as for sleep and anxiety difficulties. At a routine follow-up after heart surgery, it was noted that she was becoming anxious about medical procedures and described symptoms which were qualitatively different in their nature and intensity to past difficulties. A thorough clinical review revealed symptoms related to the build up and recovery from the most recent surgery, high physiological arousal including tearfulness, not feeling her usual self (reduced enjoyment and connectedness), cognitive avoidance of things connected to the surgery, and disrupted sleep. The intensity and duration of these, along with their level of impairment, were suggestive of PTSD, a diagnosis the young person agreed with.

Following a discussion about treatment options she agreed to start NET with her regular therapist, to aid a sense of security. Despite the therapist having worked with her for a long time, the stones she identified in her lifeline were not those that had been expected but included the psychological consequences of cancellation of a surgery at short notice and the visual image she had of herself post-surgery. The young person engaged well with the sessions and was able to work through the NET process in a structured and contained way.

The adaptations that were made were primarily around the considerations needed in a busy acute hospital setting, to ensure that clinic rooms were not too noisy, with sounds that might be triggering such as crying children. This was especially important given that the hospital was the main trigger for her and so managing the intervention within that setting added an extra level of complexity for the patient. She was taught some grounding techniques prior to the work starting along with psychoeducation about how the symptoms might at times increase in intensity.

At the end of the NET, the young person reported a significant reduction in PTSD symptoms, although these did re-emerge to a lesser extent at key anniversaries, such as of the surgery, but were more manageable. NET was a helpful way of processing the traumatic memories associated with major surgery and the lifeline helped to identify which of the many potential experiences were the ones she needed to explore.

## Discussion

This paper has described a number of clinical applications of NET that have been used to treat a range of cases of children with PTSD, some in relatively typical clinical settings and others more unusual. Although the majority of the evidence-base lies with populations who have experienced forced migration and organized violence, there are many other children and young people who have experienced multiple traumatic events who could benefit from NET. There appears to be wide applicability of NET to those affected by trauma in different settings including with co-morbid diagnoses. Therefore co-morbidity should not hinder referral for NET. Furthermore, young people who might not traditionally access PTSD treatments, such as those on the autism spectrum, or with intellectual disabilities, could engage well with NET and it should be seen as a priority and human rights imperative that these particularly vulnerable populations have access to PTSD interventions. These young people are often deemed “not ready” or “not able” to access treatment or that it might be “unsafe.” The case studies reported here and the wider literature suggests NET is appropriate and can be adapted to enable engagement and treatment (18). Furthermore, NET has demonstrated that even in contexts of ongoing difficulties and active danger, treatment can benefit those who have PTSD and that objective measures and requirements of “safety” might incorrectly hinder many from accessing or being offered treatments.

There were a number of components of common practice in all of the adaptations: the lifeline was an important part of the NET as it helped guide the number of sessions that might be needed and provided an anchor for the young person’s different traumatic exposures. Many found it helpful and some wanted to share their lifelines with key adults. Exploring the traumatic events in detail was tolerated to various extents, and all managed to complete the treatment. The treatment could be delivered in busy hospital settings as well as while pacing a corridor, it has predictable repeated questions when exploring the traumatic events which can provide comfort and containment. Many had been living with their traumatic memories for a relatively long time which suggests that improving access to effective treatments is not only necessary but possible, even if their case presentations contain specific details which may seemingly complicate progress during therapy.

Conceptualizing trauma as an event which, when experienced, destroys components of personal autobiography helps us appreciate how this approach, which includes the “lifeline” early in treatment, is important. NET has also been used by the authors with parents who have cared for physically unwell children who have needed repeated investigations. Their post-traumatic symptoms have impacted on their ability to bond with their children as well as impair their functioning. Engagement in treatment can be particularly challenging for those who have PTSD, especially for younger populations. The lifeline in some of these cases has been shown to be a potentially useful tool to facilitate engagement. It provides a relatively simple way to start to acknowledge and address past experiences, by at least naming them and placing them in chronological order. The process of NET also seems well suited to multiply traumatized populations, as no one particular memory needs to be identified early as an “index” case. All “stones” can and should be explored, as well as allowing for additional episodes to be included at later times. Sometimes the most difficult memories are not identified early in treatment as the therapeutic relationship needs to develop and the young person might not trust their therapist or believe that exploration of the event will be manageable for them. The complexity and importance of the therapeutic relationship needs to be appreciated in young people who have experienced multiple traumatic events as they are likely to have had abusive relationships with adults which can add to significant engagement challenges (46). This was discussed in one of the cases, but has been apparent, to a lesser extent, in much of the work conducted. As the therapeutic relationship develops and trust in the process increases, then young people might be better able to explore their most difficult and unmentionable experiences.

These examples give us cause for optimism. As noted by a recent meta-analysis, NET is an effective short-term therapy for reducing symptoms of PTSD and depression, particularly for war-affected refugee populations and adults (18). Although a significant proportion of those studied do not respond to treatment, for those that do, function can improve substantially. This review also noted the marked absence of studies directly comparing NET to other trauma-focused psychological interventions. Nonetheless, these case examples suggest that NET can be effective for the younger population in a wide-range of contexts with PTSD from exposure to multiple traumatic events. The applicability of NET to different situations and the relative accessibility of training in NET should be noted. The training, for example, requires 2–3 days for mental health professionals and longer (up to 2–3 weeks) for lay therapists. This makes NET a potentially scalable PTSD intervention, especially for harder to reach and more vulnerable populations where lay-therapist options, possibly through third sector organizations, might be crucial. The evidence base for NET still needs to be improved as many of the RCTs so far have been hampered by small sample sizes, non-active control groups, limited follow-up, and primarily refugees and asylum seekers studied. Furthermore, in clinical practice and research, identifying routine outcome measures that carry minimal cost and complexity as well as capturing the key symptoms and functional outcomes would dramatically facilitate improved understanding of treatment efficacy in younger populations.

NET was perceived by those included in these examples as non-threatening, although initial engagement difficulties are always likely for those with PTSD, given how prominent avoidance is for reminders of the original traumatic events. In NET the young person will be aware that they will talk about these experiences in great detail. There is a flexibility of where NET is delivered, it was originally tested in refugee camp settings, yet in these examples was conducted in a range of environments. It might even be suited to being conducted in schools where young people might find it easier to access support, raising the possibility of prevention of later psychopathology. This is aligned with the “building block effect” theory where cumulative exposure to trauma leads to increased likelihood of PTSD, therefore the role of early processing to try and prevent further harm needs to be better studied (43, 47). Exposure to potentially traumatic events might even help us understand a wider array of psychopathologies than PTSD and therefore exploration of poorly encoded memories using NET may be applicable in more contexts than just those where PTSD has fully developed. The more opportunities to study, train, and deliver treatments for PTSD, especially for child and adolescent populations is now essential, given the increasing understanding we have of the broad negative impact on child development of exposure to traumatic events and the need to make psychological treatments more accessible.

## Ethics Statement

All individuals gave written informed consent for their potentially identifiable information to be included in this article.

## Author Contributions

All authors contributed to the manuscript, were involved in writing the first draft of the paper and provided input to all stages of the paper.

## Conflict of Interest

The authors declare that the research was conducted in the absence of any commercial or financial relationships that could be construed as a potential conflict of interest.
